# Eserine and Barium Chloride in Parturient Apoplexy1Read at meeting of Michigan State Veterinary Medical Association, February 8, 1899.

**Published:** 1899-07

**Authors:** Judson Black

**Affiliations:** Richmond, Mich.


### REPORTS OF CASES.

### ESERINE AND BARIUM CHLORIDE IN PARTURIENT APOPLEXY.[1]

By Judson Black, V.S.,
RICHMOND, MICH.

At our meeting last winter the merits of eserine in the treatment of parturient apoplexy was discussed, and, as a drowning man grasps at a straw, I grasped at this hope held out for the treatment of a disease of which I had not seen one animal recover since I went into practice, and I had my share of it.

My first case after returning home I found completely comatose, lying flat on side, could give no medicine per orem, tried eserine, 1 gr., intravenously. Some peristalsis but no passage. Repeated in one hour and after the injection she moved her tail, ears, and head some but otherwise no results. Left her with unfavorable prognosis. Died a few hours afterward.

Case II. Found cow lying on sternum, head swinging, but still held up. Gave her saline purge, took her to barn on stone-boat, and when we got her fixed up comfortably she was exhibiting some coma. I gave intravenously 1 gr. eserine followed by an intravenous injection of 12 grs. Ba.Cl. in ten minutes. She arose to her feet almost instantly, trembled violently and acted crazy, became quiet in a few minutes and fell down, after which she had an evacuation. Left nux, whiskey and nit. eth., to be given every two hours. Saw her again next morning; still down. Repeated the eserine and barium chloride as before with good results (no crazy spell this time.) Repeated treatment in the evening; good results. Next morning still down but showing signs of improvement, having had a natural passage and trying to eat some. Got up before I left and made a recovery.

Case III. Found patient in about same condition as Case I. Used eserine and Ba.Cl. as in Case II. No results, except to bring her to life some; she would throw her head about and move her legs and tail, and acted as though in pain.

[1] Read at meeting of Michigan State Veterinary Medical Association, February 8, 1899.

Repeated medicine in one hour; result, a good passage. Left her with unfavorable prognosis. In the evening her condition apparently unchanged; repeated injections, with good results. The following evening somewhat improved, and as a heavy storm was coming up she was taken to the barn, when she got up on her breast and looked for something to eat, apparently. Gave a saline purge and left nux., nit. eth. and whiskey. She got up next day and made a good recovery.

Case IV. Found cow much in same condition as Case II. Treatment same, no results from injections; gradually got worse and died about forty-eight hours from the time I first saw her and about ten minutes or less after a dose of Ba.Cl., which I think hastened her end.

Case V. This case was peculiar, inasmuch as the placenta was retained, and had been removed with difficulty the day preceding the one when I was called, when a well-marked case of parturient apoplexy presented itself. She had a saline purge when the afterbirth was removed. I used the eserine and Ba.Cl. as in the preceding cases, and she recovered.

Case VI. This case I saw and treated within a month, and she got up every time she was asked to except once. She showed symptoms of this disease but not so alarming as most of them. Gave her the ordinary purgative with no results and no improvement in her cerebral symptoms. I then tried the eserine and Ba.Cl. and got peristalsis and a small passage and she improved from that time.

Such has been my experience with a disease that I had become completely discouraged with and I got so I would not go to see them all, but they died just the same.

